# Ratio of Injured Lung Volume Fraction in Prognosis Evaluation of Acute PQ Poisoning

**DOI:** 10.1155/2018/4501536

**Published:** 2018-02-13

**Authors:** JinJin Liu, Ye Xiong, Mengmeng Jiang

**Affiliations:** Department of Radiology, The First Affiliated Hospital of Wenzhou Medical University, Wenzhou, Zhejiang 325000, China

## Abstract

Although paraquat (PQ) concentrations are the most reliable prognosis predictors of PQ poisoning, these laboratory tests are not readily available in all hospitals. In this study, we proposed an imaging related parameter, that is, the ratio of injured lung volume fraction, for the prognosis evaluation of acute PQ poisoning based on the correlation between disease progress and lung imaging features. An artificial neural network was trained and then used to classify the injured and normal lung regions. The ratio of injured lung volume fraction was calculated from the injured lung volume fractions in the first and second CT scans after three-dimensional reconstruction. Parameters of blood tests were collected. A significant difference was observed with respect to the ratio of injured lung volume fraction between survivors and nonsurvivors (0.73 ± 0.17 versus 0.40 ± 0.14,* P* < 0.001). No patients survived when the ratio of injured lung volume fraction was less than 0.3, while all patients survived as the ratio of injured lung volume fraction was greater than 0.8. Thus, the ratio of injured lung volume fraction may provide an alternative and informative measure for the prognosis of acute PQ poisoning.

## 1. Introduction

As a highly effective, nonselecting, and fast-acting herbicide, paraquat (PQ) is harmless to the environment because of its rapid decomposition into nontoxic compounds after soil contact. These excellent properties led to the adoption of PQ worldwide over the past few decades. However, PQ is highly poisonous to human beings and animals and remains a primary cause of death in pesticide poisoning, especially in developing countries [1–4].

Early recognition and timely treatment play a crucial role in the survival of the patient with acute PQ poisoning. Advances in prognosis techniques would allow development of reasonable clinical treatment to help patients. To predict the survival of patients who have suffered from PQ poisoning, a variety of clinical parameters have been proposed. Plasma concentration is an extremely valuable predictor. Proudfoot et al. [5], Hart et al. [6], and Schermann et al. [7] examined plasma PQ concentrations and came up with contour graphs of plasma PQ to time relationship for different probability of survival. Sawada et al. [8], Ikebuchi et al. [9], and Jones et al. [10] reported different indices based on PQ concentration and time since ingestion to predict survival. Urine PQ concentration may also contribute to the prognosis. Scherrmann et al. [7] found that all patients with urine PQ concentration less than 1 mg/l within 24 hours of overdose survived.

However, the laboratory tests of PQ concentrations are not readily available in all hospitals; moreover, time since ingestion may not be known accurately, especially at early stage when the PQ concentrations decrease quickly, which may influence the prediction accuracy since PQ concentration is combined with time lag since ingestion to predict the prognosis. As we know, PQ poisoning may result in damage to multiple internal organs and lead to multiple organ dysfunction syndromes in severe conditions. In general, PQ mainly accumulates in the lung, which is the main target organ of PQ poisoning [11, 12]. Respiratory failure induced by acute lung injury and progressive pulmonary fibrosis is a main cause of death. Lung injury level is associated with imaging features. Im et al. [13] reported findings on chest radiography and CT in 42 patients with PQ poisoning. They found that radiographic changes during the first week after ingestion included diffuse consolidation, pneumomediastinum, and cardiomegaly with widening of the superior mediastinum. Other CT image features of lung injury in PQ poisoning at early stage, such as thickening of bronchovascular bundle and bronchiectasis, ground-glass opacity, mosaic attenuation, pulmonary consolidation, and subpleural lines, have been reported [11, 14, 15]. Thus, imaging related parameters may be used for prognosis evaluation as well.

This paper aims to study the feasibility of a CT imaging related parameter, that is, the ratio of injured lung volume fraction defined in the end of following Section 2.3, in the prognosis evaluation for patient with acute PQ poisoning.

## 2. Materials and Methods

### 2.1. Study Subjects

Imaging data was retrospectively reviewed in this paper, and this study was approved by the local institutional review board. Patients who received the first CT scan on admission and then the second CT scan within 1 week to monitor the disease progress were enrolled in this study. However, those with PQ poisoning suffered other poisoning, trauma, and/or acute diseases were excluded in this study. Fifty-eight patients with acute PQ poisoning, who were admitted to the intensive care unit within 12.6 ± 14.1 hours after ingested PQ during the period of Jan. 2014 to Dec. 2015, were finally included in this study. Of them, 32 were men and 26 were women (mean ± SD age: 39.4 ± 16.6 years). The estimated amount of ingested PQ ranged from 4 to 500 ml.

### 2.2. CT Image Acquisition

Images were acquired with a 64-channel multidetector CT scanner (Lightspeed VCT 64, GE Medical Systems, Milwaukee, WI, USA) with slice increments of 1.25 mm. X-ray tube voltage was set to 120 kV. Image data matrix was 512 × 512. Field of view ranged from 32.5 to 39.6 cm, and pixel sizes varied from 0.635 to 0.773 mm. The 1st CT scan was performed on admission and the time interval between the 1st and 2nd CT scans was 69.4 ± 24.9 hours.

### 2.3. Train of Two-Layer Feed-Forward ANN for Injured Lung Detection

**Figure 1:**
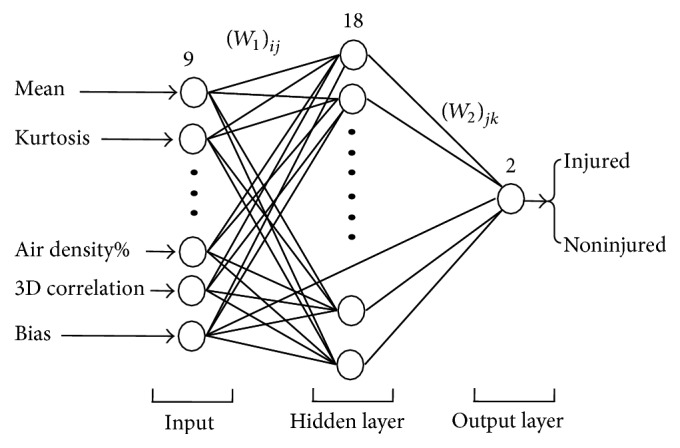
A two-layer feed-forward ANN.

A two-layer feed-forward ANN shown in Figure 1, with nonlinear sigmoid hidden and output neurons, was employed as a classifier in this study. The number of hidden units was empirically determined to be 18. For each ROI, values of texture features were calculated and used as the input data for the ANN. This ANN was applied for classifying all ROIs in the segmented lung regions into two categories, that is, injured and noninjured lung regions.

Data set in ROIs for the training of the ANN was obtained by experienced radiologists. The radiologists determined the ROIs as either injured or noninjured lung regions according to their experience, and corresponding injured or noninjured classification was set as the output of the ANN. Nine image texture features of the ROI were adopted as the input data to characterize different lung patterns, and these image features included original CT values at pixels, six statistic parameters, that is, mean, standard deviation, maximum, minimum, skewness, and kurtosis of CT values in a ROI, 3-dimensional mean CT value correlation, and fraction of the area with air density in a ROI. If part of a ROI was at the boundary of the lung and included area external to the lung, the corresponding area was ignored for image texture feature calculation. Matrix size of 7 × 7 for ROIs was chosen empirically. The fraction of the area with air density is defined as the ratio of pixel number with CT values less than −825 to the total pixel number in a ROI (i.e., 49 in this study). Assume three successive image slices *n* − 1, *n*, and *n* + 1 and corresponding CT values at pixel (*i*, *j*) are *P*_*i*,*j*_^*n*−1^,  *P*_*i*,*j*_^*n*^, and *P*_*i*,*j*_^*n*+1^, the 3-dimensional mean CT value correlation in a ROI is defined as

(1) $$\begin{array}{c} r_{co}=\left|\;\left(\;{\overset{-}{P}}_{i,j}^{n}-{\overset{-}{P}}_{i,j}^{n-1}\;\right)\left(\;{\overset{-}{P}}_{i,j}^{n}-{\overset{-}{P}}_{i,j}^{n+1}\;\right)\;\right|, \end{array}$$

where ${\overset{-}{P}}_{i,j}^{n}$ is the average CT values in a ROI at slice *n* and is defined as

(2) $$\begin{array}{c} {\overset{-}{P}}_{i,j}^{n}=\frac{1}{49}\overset{i+3,j+3}{\underset{i-3,j-3}{\sum }}P_{i,j}^{n}. \end{array}$$

The 3-dimensional mean CT value correlation is an effective parameter to classify area due to partial volume effect from injured lung regions.

**Figure 2:**
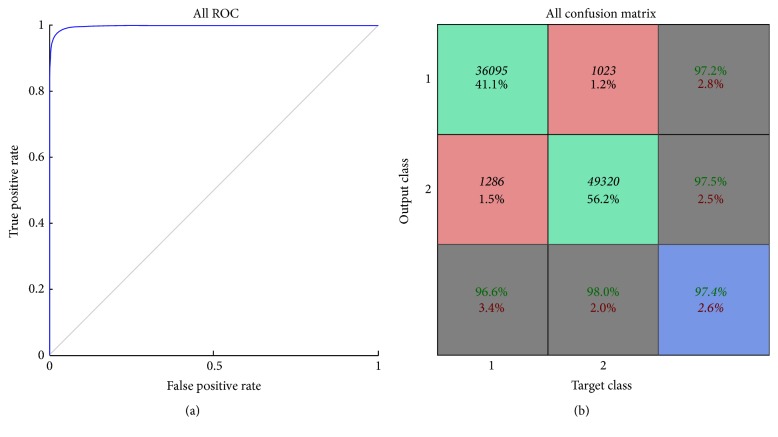
(a) Graph depicts ROC curve of ANN for classification of injured and noninjured lung ROIs. (b) Results of confusion matrix.

The total number of training data was subdivided into three groups including training, validation, and test data sets occupying 75%, 15%, and 15% of the total training data, respectively. The maximal number of iterations was limited to 5000. Receiving operating characteristic and confusion analyses were used to evaluate the performance of the ANN as shown in Figure 2, and favorable performance was observed. The trained ANN was further applied in the clinical images for the classification of injured and noninjured lung regions.

### 2.4. Calculation of Ratio of Injured Lung Volume Fraction

**Figure 3:**
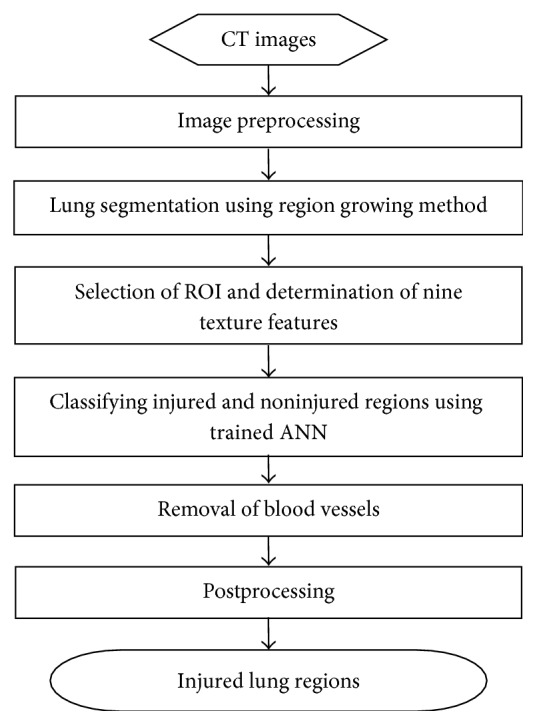
Overall image analysis procedure.

The overall image analysis procedure was demonstrated in Figure 3. CT image data in the format of Digital Imaging and Communications in Medicine (DICOM) was analyzed using a program code developed in MATLAB 7.11 software environment. To achieve effective lung segmentation, images were smoothed to reduced noise signal, whereas boundaries were preserved using anisotropic diffusion [16, 17]. Lung was segmented with region growing method including following steps: (1) identifying a seed point within the lung and close to the pleura; (2) calculating the connectivity of the points of the images to the seed point; (3) determining a 40 × 40 rectangular ROI with the seed point as center and including both lung and surrounding nonlung tissue and finding the maximum and minimum CT values within the rectangular ROI, CT_max_, and CT_min_; (4) applying the selection criteria, that is, absolute value of difference between CT values at observing point and seed point less than 0.3*∗*(CT_max_ − CT_min_), to the connectivity of the points in order to identify the extent of the lung. After lung segmentation, texture parameters at each pixel in a region of interest (ROI) in the segmented lung region were calculated. The calculated texture parameters were then used as input to the trained two-layer feed-forward artificial neural network (ANN) for classifying injured and noninjured lung regions. Blood vessels may possess similar CT values as the injured lung region. It cannot be distinguished only using the image texture features as described above. Thus, a Hessian based multiscale vessel enhancement filter [18–20] was used to segment the blood vessels, and blood vessels were then removed from the injured lung region. Finally, we removed very small regions in the segmented image.

After segmentation, whole and injured lung were imported into software package MIMICS 10.0, and corresponding 3-dimensional reconstructions were performed to calculate the volumes. The injured lung volume fraction is defined as the quotient of the injured lung volume divided by the whole lung volume; the ratio of injured lung volume fraction refers to the ratio of the injured lung volume fraction in the 1st CT scan to that in the 2nd CT scan.

### 2.5. Statistical Analysis

Continuous variables were presented as mean ± SD. Student's *t*-tests or Mann–Whitney tests were applied for continuous variables. A *χ*^2^ test was used for the gender ratio. Statistical significance was set at *P* < 0.05. All the statistical analysis was performed using IBM SPSS version 22.0 (IBM SPSS, Armonk, NY, US).

## 3. Results

**Figure 4:**
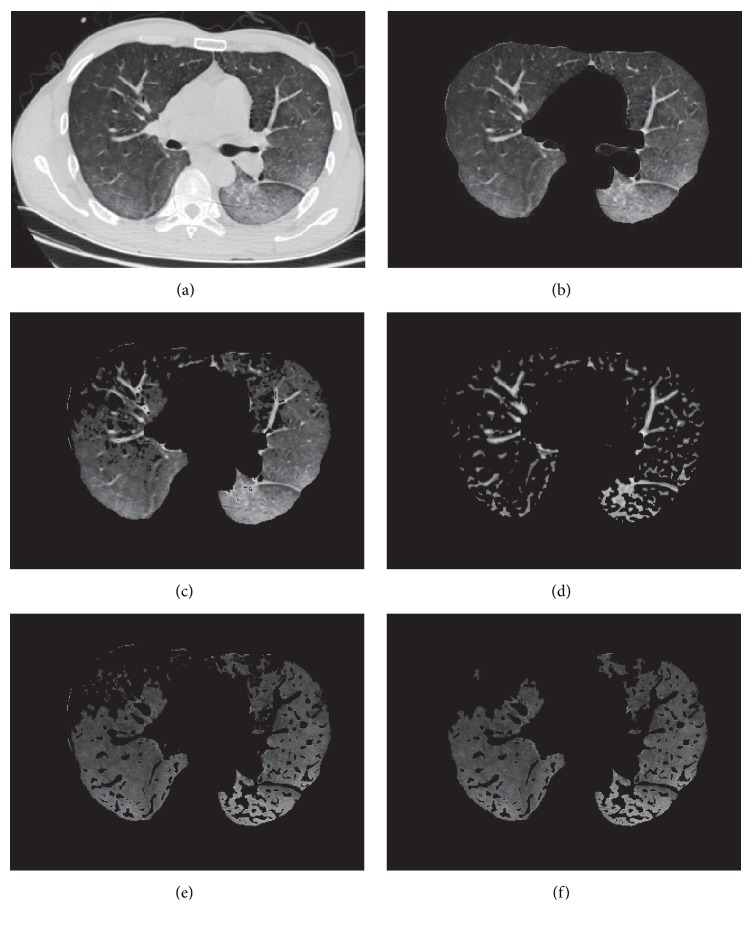
Experimental results of image analysis. (a) Original image; (b) lung segmentation; (c) candidate of injured lung region; (d) segmentation of blood vessels; (e) removal of blood vessels from candidate image; and (f) injured lung region after postprocessing.

**Figure 5:**
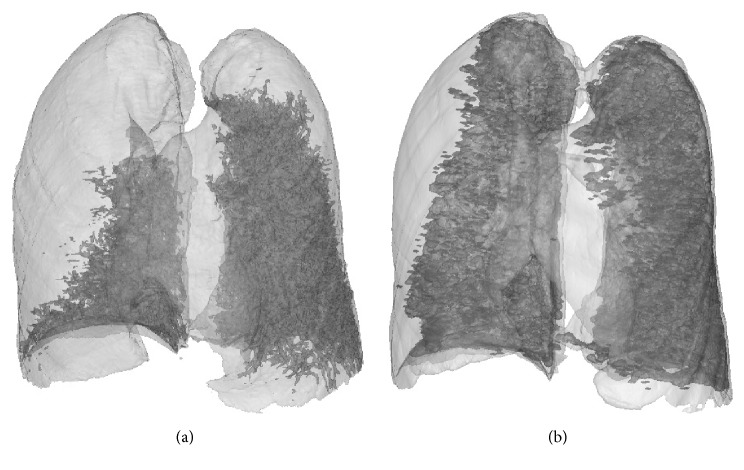
Injured lung images after 3D reconstruction at (a) 1st and (b) 2nd CT scans.

Figure 4 illustrates one of the experimental results of image analysis according to the preceding mentioned method. In Figure 4, (a) shows the original image; (b) demonstrates segmentation of lung region using region growing method; (c) shows candidate region of the injured lung classified using the trained two-layer feed-forward ANN. It was observed that some of the blood vessels were included in the candidate region; (d) illustrates blood vessels segmented using a Hessian based multiscale vessel enhancement filter; (e) presents the image of injured lung region after removal of blood vessels; and (f) shows the final results of injured lung region after postprocessing to remove very small regions. A good segmentation was achieved.  Figure 5 demonstrates three-dimensional images of injured lung tissue reconstructed from the 1st and 2nd CT scans. The injured lung volume fractions were 35.4% and 78.1%, respectively, and corresponding ratio of injured lung volume fractions was 0.45, which suggested a fast growth of injured lung area and a rapid evolution of disease. Thus, the three-dimensional images of injured lung tissue and corresponding ratio of injured lung volume fractions provided an intuitive visualization and a quantitatively evaluation for monitoring the evolution of acute lung injury caused by PQ poisoning, respectively.

Among these patients with acute PQ poisoning, 21 survived and 37 died.  Table 1 shows the comparisons of 25 parameters between survivors and nonsurvivors, including gender ratio, age, body temperature, heart rate, ratio of injured lung volume fraction, plasma PQ concentration, time lag since PQ ingestion, 4 routine blood parameters, 10 biochemical parameters, and 4 blood gas parameters. No significant differences were found between the survivors and nonsurvivors with respect to gender ratio (13 : 8 versus 19 : 18,* P* = 0.437), age (38.5 ± 17.0 versus 39.9 ± 16.6 years,* P* = 0.758), body temperature (37.0 ± 0.5 versus 36.7 ± 1.0°C,* P* = 0.251), and heart rate (84 ± 10 versus 86 ± 17 min^−1^,* P* = 0.685). As expected the plasma PQ concentration was significantly lower in survivors than in nonsurvivors (646.9 ± 1110.7 versus 22992.4 ± 36797.2 ng/mL, *P* < 0.001); there was a statistical difference between the survivors and the dead with respect to the time lag since PQ ingestion (16.9 ± 17.7 versus 10.1 ± 11.1 h,* P* = 0.038). For 4 routine blood parameters, white blood cell (WBC) and red blood cell (RBC) were lower in survivors than in nonsurvivors (12.74 ± 5.50 versus 20.41 ± 10.61 ×10^9^/L*, P* = 0.001 for WBC; 4.45 ± 0.49 versus 4.84 ± 0.63 ×10^12^/L,* P* = 0.018 for RBC), while no significant difference was found in hemoglobin (Hb) and blood platelet count (BPC) between survivors and nonsurvivors. For 10 biochemical parameters, there were significant differences between survivors and nonsurvivors with respect to aspartate aminotransferase (AST) (24 ± 12 versus 107 ± 145 U/L,* P* < 0.001), alanine aminotransferase (ALT) (24 ± 25 versus 67 ± 102 U/L,* P* = 0.008), total bilirubin (15 ± 7 versus 28 ± 30 *µ*mol/L,* P* = 0.023), glucose (6.5 ± 1.9 versus 8.5 ± 3.5 mmol/L,* P* = 0.005), and potassium (3.60 ± 0.42 versus 3.31 ± 0.48 mmol/L,* P* = 0.027); however, no statistical differences were observed in ALT/AST ratio, urea nitrogen, creatinine, sodium, and chloride between the survivors and the death. As to 4 blood gas parameters, significant difference was found in carbon dioxide partial pressure (PCO_2_) between survivors and nonsurvivors (31.7 ± 5.6 versus 36.8 ± 5.8 mmHg,* P* = 0.002); HCO_3_ was significantly larger in survivors than in nonsurvivors (22.3 ± 2.7 versus 18.2 ± 4.5 mmol/L,* P* < 0.001); no statistical differences were found in pH and oxygen partial pressure (PO_2_) between survivors and nonsurvivors.

**Figure 6:**
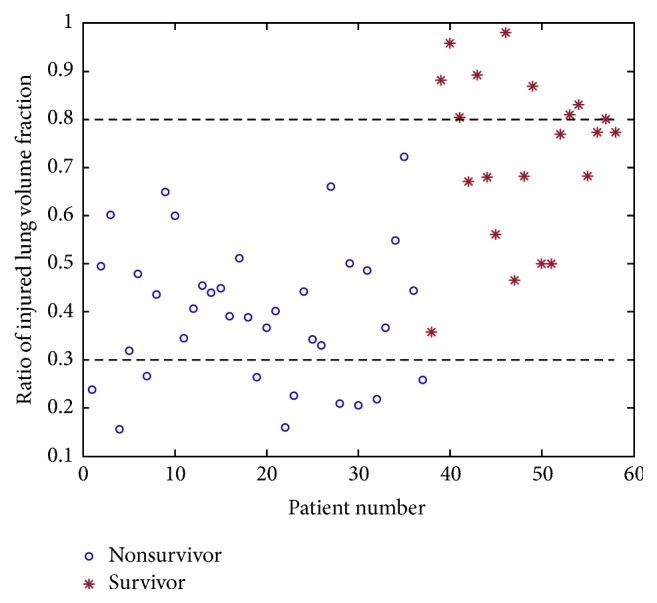
A scatter plot of ratio of injured lung volume fraction for both survivors and nonsurvivors.

The ratio of injured lung volume fraction was 0.73 ± 0.17 in survivors and 0.40 ± 0.14 in those who did not survive (*P* < 0.001, Table 1). A scatter plot of the ratio of injured lung volume fraction for both survivors and nonsurvivors is demonstrated in Figure 6. Two black level lines in Figure 6 implied that no patients survived when the ratio of injured lung volume fraction was less than 0.3; by contrast, all patients survived as the ratio of injured lung volume fraction was greater than 0.8.

## 4. Discussion 

In this study, the ratio of injured lung volume fraction was applied for the prognosis evaluation in acute PQ poisoning. Results showed that the ratio of injured lung volume fraction was closely correlated with prognosis of acute PQ poisoning and might be used to monitor the disease progress. The injured lung region in CT images was classified using a trained two-layer feed-forward ANN. The ANN provided a valuable tool to segment the injured lung region and greatly improved the efficiency of segmentation. After segmentation, the injured lung and whole lung were reconstructed and corresponding volumes were calculated from the three-dimensional images. The three-dimensional visualization of injured lung and corresponding ratio of injured lung volume fraction provided us with an intuitive visualization and quantitative evaluation for the prognosis of acute PQ poisoning, respectively.

Qualitative and semiquantitative laboratory tests in a great variety of biological fluids and tissues, such as plasma, urine, gastric dialysate, and aspirate, are feasible to predict the prognosis of acute PQ poisoning. Numerous parameters from laboratory tests, including plasma PQ concentration, urine PQ concentration, blood biochemistry, arterial blood gases, and complete blood count, have been investigated to predict the prognosis of acute PQ poisoning. Quite a few of these tests are not readily available in all hospitals. In addition to these laboratory measurements, supplementary information of medical images may provide valuable information for the prognosis evaluation. Image observations from medical modalities, such as CT and DR, have revealed the strong correlation between imaging features and disease progress [11, 13–15]. Kim et al. [21] believed that lung injury was the main cause of death after PQ poisoning and lung abnormalities might be a better predictor. They investigated the feasibility of high resolution CT of lungs in prognosis of acute PQ poisoning and found that the area of ground-glass opacities in the lung was a useful predictor of survival in acute PQ poisoning, especially in patients with low plasma PQ levels. Their finding is valuable because although PQ levels are proven to be an excellent predictor, numerous patients still died at low PQ levels according to their previous analysis. However, regions of ground-glass opacity in their work were recognized by radiologists and corresponding area was manually measured. The process was time-consuming. Different radiologists may have different judgments for ground-glass opacity. Second, in addition to ground-glass opacity, other imaging characteristics, such as consolidation and mosaic attenuation, may also contribute to lung injury. Moreover, area is 2-dimensional information and area of GGO in which slice is useful for prediction is difficult to be determined. Three-dimensional volume information may provide more useful information in prognosis prediction. Kang et al. [22] used the volume ratio of GGO in early lung CT to predict mortality in acute PQ poisoning. They found that the volume ratio of GGO was reliable and independent predictors of outcome in acute PQ poisoning and achieved high sensitivity of 85.4% and specificity of 89.3%. In this study, another image related parameter, that is, ratio of injured volume fraction, was used for prognosis evaluation. An automatic segmentation algorithm was developed. A trained ANN was applied to eliminate subject judgments of injured lung region at each slice. The reconstructed injured lung volume after segmentation presented an intuitive visualization of disease progress.

It is necessary to point out that we did not try to prove that the ratio of injured lung volume fraction was superior to other laboratory parameters in the prognosis prediction of acute PQ poisoning, such as the plasma or urine PQ concentrations. Instead, the new proposed imaging related parameter in this article provides a complementary, alternative, and informative measure for the prognosis analysis of acute PQ poisoning, especially when the plasma or urine PQ concentrations are not readily available in some hospitals.

There are several limitations in this study. First, all study subjects came from a single center and a small number of patients were enrolled. Currently, there remains no standardized and effective treatment method for PQ poisoning. The survivor rate is medical center dependent, which may influence analysis results. Second, these patients were often scanned under free breathing, and respiratory motion might cause artifacts in CT scanning, which might influence the calculation of injured lung volume. Moreover, the output of ANN is required to be known during the training stage of ANN in order to enable ANN to learn from the observing data sets and account for complicated nonlinear relationships between input and output. In this study, output of ANN (i.e., the injured and noninjured lung regions) was determined by experienced radiologists during the training stage of ANN, and this may bring subjective disturbance in the analysis. Finally, injured lung volume fraction was time-dependent; however, it was impossible to continuously scan the lung of a patient to observe the development of injured lung volume fraction due to radiation and economic issue. This was a retrospective study and there was no standardized second scan time. Time interval between the 1st and 2nd CT scans varied among patients, which might influence on the clinic application of the ratio of injured lung volume fraction.

## 5. Conclusions

In conclusion, with region growing segmentation method and an ANN, an automatic scheme was proposed to calculate the injured lung volume. In addition to general laboratory tests, the ratio of injured lung volume fraction may be applied for prognosis evaluation of acute PQ poisoning, especially when PQ concentrations are not available. Our study may aid in the development of reasonable clinical treatment to help patients.

## Figures and Tables

**Table 1:** Comparisons of parameters between the survivors and the death.

Parameters	Survivor (*n* = 21)	Nonsurvivor (*n* = 37)	*P* value
Ratio of injured lung volume fraction	0.73 ± 0.17	0.40 ± 0.14	<0.001
Gender ratio (male : female)	13 : 8	19 : 18	0.437
Age	38.5 ± 17.0	39.9 ± 16.6	0.758
Body temperature (°C)	37.0 ± 0.5	36.7 ± 1.0	0.251
Heart rate (beats/min^−1^)	84 ± 10	86 ± 17	0.685
Plasma PQ concentration (ng/mL)	646.9 ± 1110.7	22992 ± 36797.2	0.008
Time lag since PQ ingestion (h)	16.9 ± 17.7	10.1 ± 11.1	0.038
WBC (×10^9^/L)	12.74 ± 5.50	20.41 ± 10.61	0.001
RBC (×10^12^/L)	4.45 ± 0.49	4.84 ± 0.63	0.018
Hb (g/L)	135 ± 15	143 ± 22	0.176
BPC (×10^9^/L)	206 ± 73	224 ± 74	0.384
AST (U/L)	24 ± 12	107 ± 145	<0.001
ALT (U/L)	24 ± 25	67 ± 102	0.008
ALT/AST ratio	0.88 ± 0.44	0.66 ± 0.38	0.072
Total bilirubin (*µ*mol/L)	15 ± 7	28 ± 30	0.023
Glucose (mmol/L)	6.5 ± 1.9	8.5 ± 3.5	0.005
Urea nitrogen (mmol/L)	4.7 ± 2.1	5.7 ± 5.6	0.443
Creatinine (*µ*mol/L)	66 ± 20	102 ± 112	0.067
Serum potassium (mmol/L)	3.60 ± 0.42	3.31 ± 0.48	0.027
Serum sodium (mmol/L)	140 ± 5	142 ± 4	0.053
Serum chloride (mmol/L)	104 ± 4	104 ± 4	0.57
pH	7.401 ± 0.043	7.368 ± 0.100	0.174
PCO_2_ (mmHg)	31.7 ± 5.6	36.8 ± 5.8	0.002
PO_2_ (mmHg)	94.0 ± 16.4	96.2 ± 21.2	0.699
HCO_3_ (mmol/L)	22.3 ± 2.7	18.2 ± 4.5	<0.001

## References

[1] Klein-Schwartz W., Smith G. S. (1997). Agricultural and horticultural chemical poisonings: Mortality and morbidity in the United States. *Annals of Emergency Medicine*.

[2] Gawarammana I. B., Buckley N. A. (2011). Medical management of paraquat ingestion. *British Journal of Clinical Pharmacology*.

[3] Eddleston M. (2000). Patterns and problems of deliberate self-poisoning in the developing world. *QJM*.

[4] Mowry J. B., Spyker D. A., Brooks D. E., Mcmillan N., Schauben J. L. (2015). 2014 annual report of the American association of poison control centers National Poison Data System (NPDS): 32nd Annual Report. *Clinical Toxicology*.

[5] Proudfoot A. T., Stewart M. S., Levitt T., Widdop B. (1979). Paraquat poisoning: significance of plasma-paraquat concentrations. *The Lancet*.

[6] Hart T. B., Nevitt A., Whitehead A. (1984). A new statistical approach to the prognostic significance of plasma paraquat concentrations. *The Lancet*.

[7] Scherrmann J. M., Houze P., Bismuth C., Bourdon R. (1987). Prognostic value of plasma and urine paraquat concentration. *Human & Experimental Toxicology*.

[8] Sawada Y., Yamamoto I., Hirokane T., Nagai Y., Satoh Y., Ueyama M. (1988). SEVERITY INDEX OF PARAQUAT POISONING. *The Lancet*.

[9] Ikebuchi J., Proudfoot A. T., Matsubara K. (1993). Toxicological index of paraquat: A new strategy for assessment of severity of paraquat poisoning in 128 patients. *Forensic Science International*.

[10] Jones A. L., Elton R., Flanagan R. (1999). Multiple logistic regression analysis of plasma paraquat concentrations as a predictor of outcome in 375 cases of paraquat poisoning. *QJM*.

[11] Dinis-Oliveira R. J., Duarte J. A., Sánchez-Navarro A., Remião F., Bastos M. L., Carvalho F. (2008). Paraquat poisonings: mechanisms of lung toxicity, clinical features, and treatment. *Critical Reviews in Toxicology*.

[12] Smith L. (1982). Young Scientists Award lecture 1981: the identification of an accumulation system for diamines and polyamines into the lung and its relevance to paraquat toxicity. *Archives of Toxicology*.

[13] Im J.-G., Lee K. S., Han M. C., Kim S. J., Kim I. O. (1991). Paraquat poisoning: Findings on chest radiography and CT in 42 patients. *American Journal of Roentgenology*.

[14] Huh J. W., Hong S. B., Lim C.-M., Do K.-H., Lee J. S., Koh Y. (2006). Sequential radiologic and functional pulmonary changes in patients with paraquat intoxication. *International Journal of Occupational Medicine and Environmental Health*.

[15] Sang Hun Lee (1995). Paraquat poisoning of the lung: Thin-section CT findings. *Radiology*.

[16] Whitaker R. T., Pizer S. M. Geometry-based image segmentation using anisotropic diffusion.

[17] Perona P., Malik J. (1990). Scale-space and edge detection using anisotropic diffusion. *IEEE Transactions on Pattern Analysis Machine Intelligence*.

[18] Frangi A. F., Niessen W. J., Vincken K. L., Viergever M. A. Multiscale vessel enhancement filtering.

[19] Campbell I. C., Coudrillier B., Mensah J., Abel R. L., Ethier C. R. (2015). Automated segmentation of the lamina cribrosa using Frangi's filter: A novel approach for rapid identification of tissue volume fraction and beam orientation in a trabeculated structure in the eye. *Journal of the Royal Society Interface*.

[20] Shikata H., Huffman E. A., Sonka M. Automated segmentation of pulmonary vascular tree From 3D CT images.

[21] Kim Y.-T., Jou S.-S., Lee H.-S. (2009). The area of ground glass opacities of the lungs as a predictive factor in acute paraquat intoxication. *Journal of Korean Medical Science*.

[22] Kang X., Hu D.-Y., Li C.-B. (2015). The volume ratio of ground glass opacity in early lung ct predicts mortality in acute paraquat poisoning. *PLoS ONE*.

